# An unusual foreign body migrating through time and tissues

**DOI:** 10.1186/1746-160X-2-30

**Published:** 2006-09-11

**Authors:** Basile N Landis, Roland Giger

**Affiliations:** 1 Service d'ORL et de Chirurgie cervico-faciale, Hôpitaux Universitaires de Genève, Switzerland

## Abstract

**Background:**

Beside infections, foreign body incidences are amongst the most frequently encountered pathologies in pediatric otolaryngology. While inhaled foreign bodies represent an acute emergency, symptoms of ingested foreign bodies sometimes appear with some delay. Typically fishbones tend to go unnoticed in a first examination and become symptomatic by fever, odynodyspahgia and torticollis. Exceptionally, foreign bodies migrate and become manifest with a considerable delay.

**Case report:**

We present a case of a young girl who presented with an unusual foreign body which migrated through the cervical tissues causing repeated cervical tumescence's before being diagnosed.

**Conclusion:**

Repeated cervical abscesses or tumescence's in children or young patients should alert the treating physician to seek for an underlying pathology such as unnoticed foreign bodies or malformations (e.g. cysts). Further the scarce literature on these migrating foreign bodies is discussed.

## Background

The most frequent ingested foreign bodies in the Ear Nose and Throat sphere are chicken and fish bones [1]. The symptoms are immediate and patients quickly seek for medical help after a few unsuccessful trials to extract the foreign body by themselves. Beside the tonsils, the base of the tongue and the upper esophagus are the places where usually the impacted foreign bodies are found [1]. Their removal is essential to prevent super-infections, abscesses and perforations with potentially life threatening mediastinal complications in case of esophageal foreign bodies [2]. Although rarely, foreign bodies sometimes migrate within the tissues and become symptomatic after a certain time lapse [3]. In those cases, the direct relation between the suspected foreign body ingestion and the first symptoms is rarely established due to the latency and unusual clinical presentation [4,5].

## Case report

**Figure 1:**
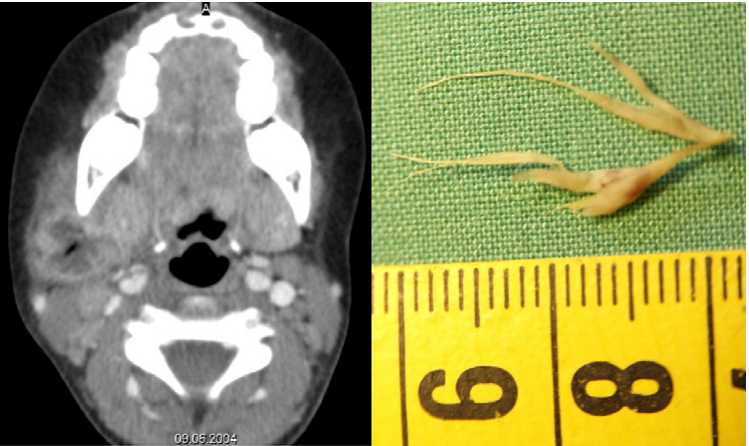
a: Computed tomography (CT) of the cervical abscess. b: Extracted foreign body. A grass blade of 2 cm of length.

We report the case of a 4-year old girl who was admitted to our ENT outpatient clinic with a cervical neck mass without other signs and symptoms. The patients history revealed, that she had previously been treated several times for odynophagia with cervical tumescence within the last two month. Symptoms and swelling disappeared temporally after the antibiotic treatments. However, the cervical mass rapidly reappeared after the end of the treatment. Otolaryngological examination showed no particularity, beside a firm lateral cervical mass. A cervical CT scan (Fig 1a) revealed a deep subcutaneous collection, suggesting the presence of an cervical abscess. Potential infectious origins such as the tonsils, the salivary glands, teeth or the facial skin were calm. Despite an intravenous antibiotic treatment with decrease of the cervical mass, an ultrasound control 10 days later showed a persistent subcutaneous liquid collection. We then opted for incision and drainage of this collection. The drainage and cleaning of the abscess cavity unearthed a blade of grass within the purulent discharge (Fig 1b).

Reviewing the patients history, the parents suddenly recalled she had complained of a transitory foreign body feeling during several days after chewing a blade of grass two months ago. Follow-up showed no further recurrence of the neck swelling.

## Discussion

Ingested foreign bodies (FB) in children vary in shape and size, whereas coins, nonmetallic sharp objects and other blunt objects seem to be the favorite items (for a detailed overview see [6]). A majority of ingested FB pass trough the gastrointestinal tract uneventfully. Severe complications are rare and often associated with delayed discovery due to silent and protracted clinical manifestations such as new onset asthma, excessive salivation or recurrent upper respiratory infections [3]. These undetected FB tend to create fistulas to the surrounding structures (e.g. aorta, bronchia, etc.) leading to potential life-threatening situations [3]. In contrast to adults, where symptoms and information on the swallowed object facilitates the diagnostic and therapeutic approach, children often present with few or absent symptoms and absence of symptoms does not preclude the presence of a FB [6]. However the detection of a foreign body and the follow-up of the clinical course is crucial, especially since complications even sometimes occur after it has been extracted [7].

Impacted foreign bodies within the ENT sphere, typically fish bones, have been reported to cause upper respiratory airway tract abscesses [8]. However, the migration through the entire pharyngeal wall ending in a superficial cervical abscess several months later is uncommon but has to be considered [1,5,9,10]. Repeated abscesses which seem resistant to treatment should always evoke the possibility of a foreign body or an underlying congenital malformation such as branchial cleft cysts [8], even if radiological examination fails to evidence its presence. While FB migration has been reported in adults [1,9], the present case reports this rare complication in a child. Particularly, the FB's nature – a grass blade – seems uncommon, even amongst adult reports [9]. Even though a glass blade is not solid or hard, depending on the ingestion angle, it can exhibit a considerable sharpness. In the present case this might have facilitated the initial tissue penetration.

Similar to foreign bodies in the ear [11] or nose [12], ingested FB in children are prone to lead to chronic and delayed symptoms [3]. Thus the possibility of a ingested foreign body should always been considered even when initial investigations where negative.
